# The role of primary intraocular lens implantation in the risk of secondary glaucoma following congenital cataract surgery: A systematic review and meta-analysis

**DOI:** 10.1371/journal.pone.0214684

**Published:** 2019-04-01

**Authors:** Shuo Zhang, Jiaxing Wang, Ying Li, Ye Liu, Li He, Xiaobo Xia

**Affiliations:** 1 Department of Ophthalmology, Xiangya Hospital, Central South University, Changsha, Hunan, China; 2 Xiangya Eye Center, Central South University, Changsha, Hunan, China; 3 Department of Ophthalmology, Emory University, Atlanta, Georgia, United States of America; 4 Department of Health Care Organization and Policy, School of Public Health, University of Alabama at Birmingham, Birmingham, Alabama, United States of America; 5 Department of Medicine, University of Alabama at Birmingham, Birmingham, Alabama, United States of America; Wenzhou Medical University, CHINA

## Abstract

**Objective:**

This meta-analysis aims to evaluate the incidence of secondary glaucoma in patients under the age of 2 years who underwent congenital cataract surgery with or without primary intraocular lens (IOL) implantation.

**Methods:**

An electronic literature search was performed in Medline, EMBASE, and Web of Science databases to retrieve studies between January 2011 and November 2018. Patients with congenital cataract who did primary IOL implantation, aphakia, or secondary IOL implantation followed by receiving extraction surgery were included in this study. Relevant studies meeting defined eligibility criteria were selected and reviewed systematically by meta-analysis. Long-term incidences of secondary glaucoma, which developed at least one year after cataract surgery, were considered and discussed as clinical outcomes in each cohort. The pooled data were analyzed according to a random effects model.

**Results:**

Eight publications involving 892 eyes were included in the current meta-analysis. In the general population of eyes with congenital cataract, the long-term incidence of secondary glaucoma was lower (P = 0.06) in eyes with primary IOL (9.5%) than in eyes without primary IOL (15.1%), including aphakia and secondary IOL. The pooled risk ratio (RR) favors primary IOL implantation in all patients (RR = 0.63). For bilateral congenital cataract, the incidence was 6.7% in eyes with primary IOL implantation, which is significantly lower than the 16.7% in eyes with aphakia and secondary IOL implantation (P<0.05, RR = 0.44). However, for unilateral congenital cataract surgery, the incidence was very similar in eyes with and without primary IOL (12.4% vs 12.0%, P = 0.61, RR = 0.87).

**Conclusions:**

In patients under 2 years of age, primary IOL implantation for bilateral congenital cataract surgery is associated with a lower risk of secondary glaucoma.

## Introduction

Cataract is a common ocular disease characterized by the opacification of the crystalline lens. Based on etiology, cataracts can be divided into several categories: congenital, age-related, metabolic, drug-induced, toxic, and traumatic.

Congenital cataract is mainly caused by genetic or environmental factors, such as gene mutation or infection with rubella virus during early pregnancy [1]. It usually presents at birth and can be diagnosed by routine examination or is noticed in connection with leukocoria or strabismus. Congenital cataract is the most prevalent treatable cause of blindness and visual impairment in children; worldwide, there are 20,000–40,000 children born with congenital cataract each year [2,3]. Therefore, appropriate, effective, and timely surgery in children with congenital cataract is highly desirable, because it can reduce the rate of blindness and improve vision in young populations.

The timing of cataract surgery is critical for children with congenital cataract. According to the majority of published studies, early surgical intervention should be performed to avoid vision deprivation during the sensitive and critical period of vision development [4]. Importantly, there are several options to correct aphakia following cataract surgery for congenital cataract, including primary intraocular lens (IOL) implantation, the use of spectacles or contact lenses, or secondary IOL implantation.

Cataract surgery and IOL implantation performed at the same time, also known as primary IOL implantation, may increase the risk of myopia shift [5,6], intense posterior capsulate opacification, and excessive inflammation because of ocular growth and axial length elongation. However, this option can reduce the anesthesia duration and the surgery expenses. The second option is to remain in an aphakic status immediately after the lens excision and undergo IOL implantation several months or several years afterwards and/or to wear contact lens to correct amblyopia. Studies have shown that contact lenses or secondary IOL implantation after surgery are effective treatment options for aphakia in children, despite several disadvantages. For example, contact lenses have the drawback of inconvenience and non-compliance in infants and children, whereas secondary IOL implantation can cause several complications, including secondary membrane formation, IOL decentration, or secondary glaucoma [7].

With the development of surgical techniques and biomaterials in recent decades, most pediatric ophthalmologists reached the consensus that primary IOL implantation is valid and safe for children above the age of 2 years. However, considering the pros and cons of primary IOLs, the ocular anatomical factors in children, and the postoperative complications, it is still controversial which surgical option to choose for children with congenital cataract under the age of 2 years. Furthermore, both primary and secondary IOL implantation carry a risk of complications, so reducing the incidence of surgical complication is another crucial concern for surgeons. At present, secondary glaucoma remains the most common complication threatening the vision after modern cataract surgery and may cause irreversible vision loss [8]. To provide evidence-based recommendations for healthcare professionals facing the same problems regarding the optimal therapeutic strategy and to provide a reference for the clinical practice, we conducted meta-analyses to evaluate the incidence of secondary glaucoma in (1) patients after primary IOL implantation, (2) patients with remaining aphakia, and (3) patients with secondary IOL implantation after cataract surgery.

## Methods

This article was composed following the Preferred Reporting Items for Systematic Reviews and Meta-Analyses (PRISMA) guidelines (checklist is shown in S1 Table) [9].

### Search strategy

Studies comparing congenital cataract treatment with or without primary IOL implantation were identified by a literature search in the following databases: Medline/PubMed, EMBASE/Ovid, and Web of Science. Searches were performed in November 2018, using “congenital cataract”, “primary intraocular lens implantation”, “secondary intraocular lens implantation”, “glaucoma”, and “children” as key phrases in various combinations. We did not restrict our search to specific languages. Furthermore, we conducted a manual search of the reference lists from each identified article to acquire additional related studies or references.

### Selection criteria

Selected trials fulfilling the following inclusion criteria were included in the analysis: (1) patients with a confirmed diagnosis of congenital cataract; (2) patients who underwent cataract extraction surgery; (3) age at cataract extraction was below 2 years; (4) the study compared cataract surgery with and without primary IOL implantation; (5) prospective or retrospective design; and (6) development of secondary glaucoma as the primary outcome measure.

Exclusion criteria were as follows: (1) patients with a history of ocular diseases other than congenital cataract, especially congenital glaucoma or any form of corneal pathology; (2) patients with a history of intraocular surgery other than cataract extraction and IOL implantation; (3) patients with syndromes like congenital rubella syndrome or Marfan syndrome, which can increase the incidence of ocular diseases; (4) prematurity (<36 weeks of gestation); and (5) follow-up time less than one year[10].

### Data extraction

Shuo Zhang (primary reviewer) and Ying Li (secondary reviewer) searched independently according to the above criteria and retrieved full-text versions of all potentially eligible studies after screening titles and abstracts of each publication. All disagreements were resolved consensually after discussions. In cases where studies provided limited information on the intervention or postoperative outcome, authors were contacted to provide additional data in detail. All items below were collected from each publication: author’s name, year of publication, design of the study, group size, age at cataract extraction, therapeutic interventions (primary IOL, aphakia, or secondary IOL), secondary glaucoma (number or rate), and follow-up duration.

### Outcome measures

The outcome measure was defined as the long-term secondary glaucoma rate, which means glaucoma developed at least one year after cataract surgery. The diagnosis criteria of secondary glaucoma from included studies varies, most of which made the diagnosis by elevated IOP (over 21mmHg) in 2 to 3 random tests plus one or more of the following combination signs: (1) optic disc cupping ≥0.3/ asymmetry ≥0.2/progression, (2)corneal changes, (3)progressive myopia shift [11–17], while the other study diagnosed glaucoma only if the patients with elevated IOP tested 2 to 3 times randomly [18]. If there was more than one published report on the same population or group of patients, the most recent results with complementary data from previous articles were used for the statistical analysis.

### Statistics and meta-analysis

Data are presented as numbers or rates. Forest plots were performed using the software R version 3.5.1 (R Foundation for Statistical Computing, Vienna, Austria) and the R package “meta” [19].

We selected the incidence of secondary glaucoma as the outcome, and the relative risk ratios (RRs) and 95% confidence intervals (CIs) of the results were compared. Due to the high likelihood of heterogeneity among the selected randomized trials and observational studies, we used a random effects model to evaluate pooled effects. Heterogeneity between studies was assessed using the I^2^ statistic and the χ^2^ test [20,21]. Finally, publication bias was calculated using the Egger test (P>0.05 was considered no publication bias) and assessed visually with funnel plots [22,23]. A value of P<0.05 was considered statistically significant.

## Results

### Search results

**Fig 1:**
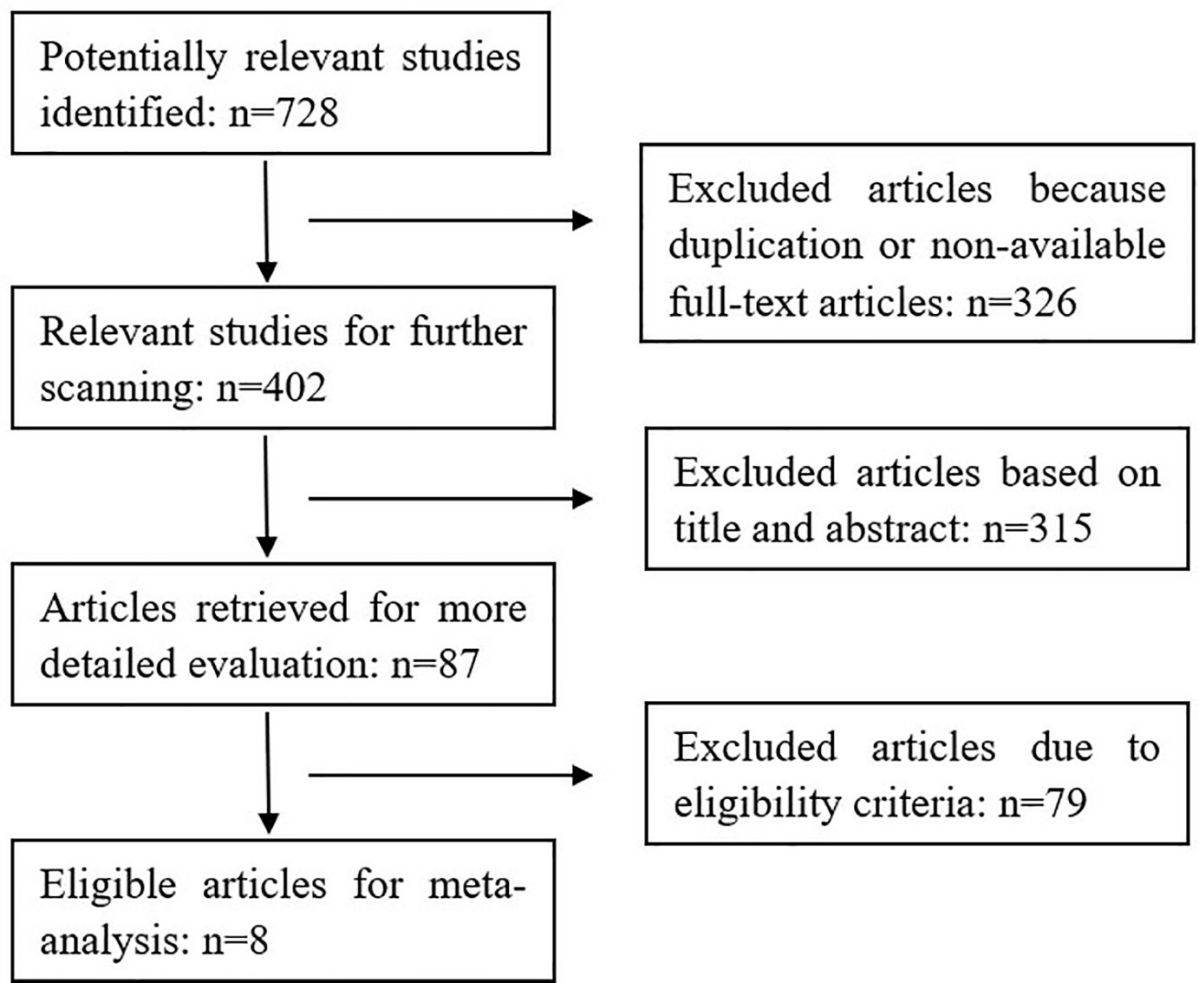
Flow chart of the literature search and the study selection process for the meta-analysis.

The results of our search strategy are shown in Fig 1. Potentially relevant publications were identified through literature search from multiple databases before November 2018. Based on a quick scan of article titles and abstracts, we identified 87 articles as potential targets for detailed evaluation. After the assessment of the eligibility criteria in detail, eight publications with a total of 892 eyes were included in the final meta-analysis. Details of these eight articles are summarized in Table 1. No statistical differences were found regarding the age at cataract extraction or the patients’ sex.

**Table 1:** Characteristics of studies included in the meta-analysis.

Author, year	No. of patients	Primary IOL Aphakia Secondary IOL	Bilateral/unilateral	Mean age at cataract extraction (months)	Mean time of follow-up (years)	Bilateral/unilateral long-term incidence of secondary glaucoma: numbers (percentages)
Vasavada AR, 2018 [11]	108	58	58/-	8.1±5.0	5.0	7/- (12.1%/-)
		50	50/-	6.3±4.2	5.0	8/- (16.0%/-)
		-	-	-	-	-
Tadros D, 2016 [12]	26	13	-/13	2.7±2.2	10.5±4.2	-/2 (-/15.4%)
		-	-	-	-	-
		13	-/13	1.9±1.8	8.6±3.0	-/3 (-/23.1%)
Freedman SF, 2015 [13]	114	57	-/57	0.9–3.9	4.4–5.3	-/11 (-/19.3%)
		57	-/57	0.9–3.9	4.4–5.3	-/9 (-/15.8%)
		-	-	-	-	-
Solebo AL, 2015 [14]	221	104	56/48	0.8–23.3	1	5/3 (8.9%/6.3%)
		117	75/42	0.5–19.0	1	21/4 (28.0%/9.5%)
		-	-	-	-	-
Li Q, 2014 [15]	60	30	-/30	12.0–24.0	1	-/3 (-/10.0%)
		30	-/30	12.0–24.0	1	-/1 (-/3.3%)
		-	-	-	-	-
Zhang H, 2013 [18]	204	47	41/6	21.2±9.0	2.0±1.6	0/0 (0%/0%)
		71	62/9	9.9±7.5	1.5±0.9	2/0 (3.2%/0%)
		86	75/11	9.9±7.5	0.9±0.6	13/0 (17.3%/0%)
Magli A, 2013 [17]	66	30	30/-	6.8±4.2	1.5±1.2	1/- (3.3%/-)
		-	-	-	-	-
		36	36/-	5.4±2.8	2.1±0.7	0/- (0%/-)
Kirwan C, 2010 [16]	93	49	10/39	<2.5	1.3±0.8	0/5 (0%/12.8%)
		44	31/13	<2.5	2.9±1.3	11/4 (35.5%/30.8%)
		-	-	-	-	-

### Outcomes of primary IOL in all patients

**Fig 2:**
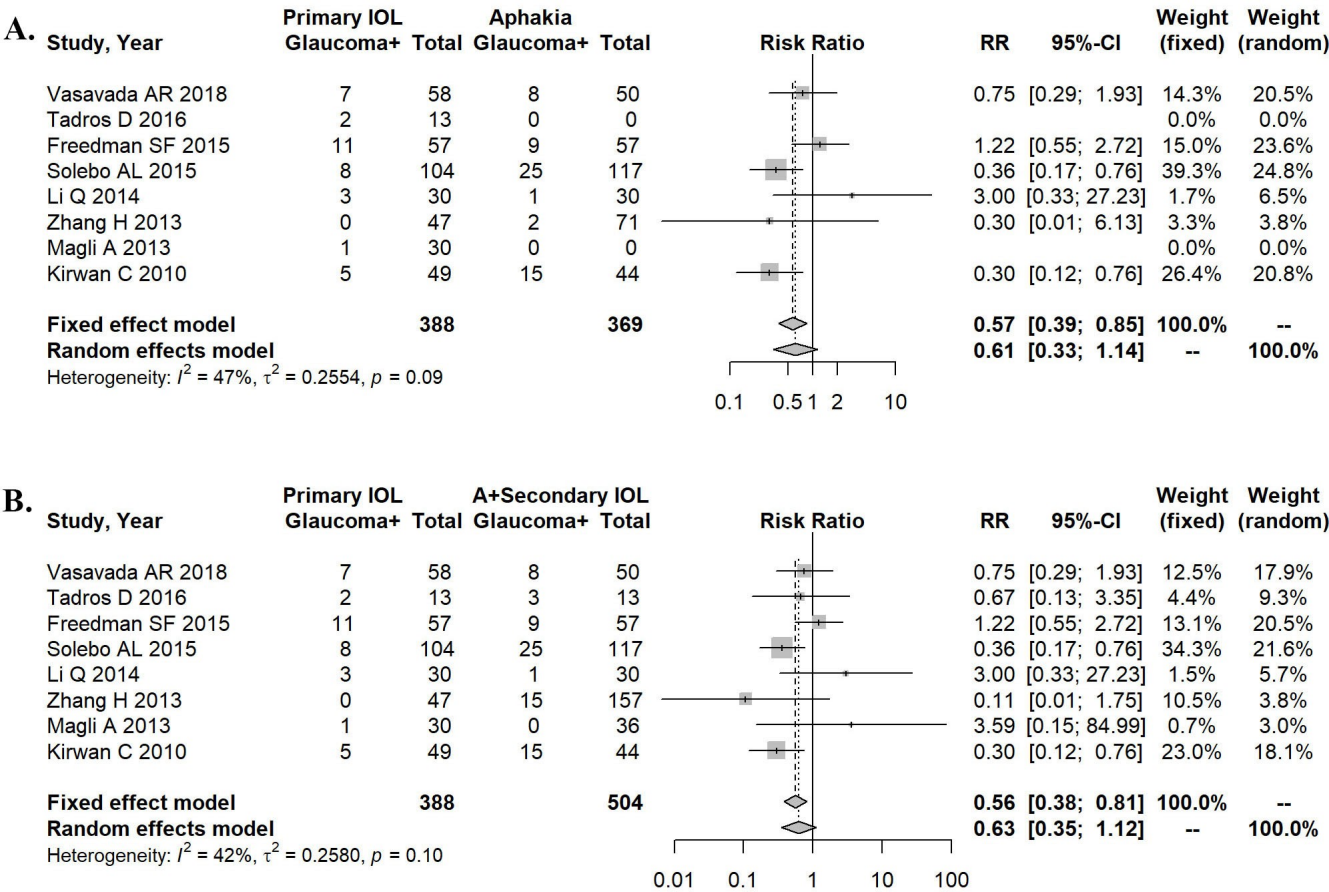
Forest plots describing the treatment effects in all congenital cataract cases. A. Development of secondary glaucoma after primary IOL implantation versus aphakia. B. Primary IOL implantation versus aphakia and secondary IOL implantation regarding the development of secondary glaucoma.

When comparing primary IOL with aphakia only, the heterogeneity of effect size was 47% (P = 0.09). A random effects model was used for the meta-analysis (Fig 2A). The result of this analysis favored primary IOL (RR = 0.61, 95% CI: 0.33–1.14). No statistically significant difference was found in the incidence of postoperative glaucoma between the two conditions (P = 0.063).

When comparing primary IOL with aphakia and secondary IOL, the heterogeneity of effect size was 42% (P = 0.10). The random effects model used for the meta-analysis (see Fig 2B) favored again primary IOL (RR = 0.63, 95% CI: 0.35–1.12). However, the incidence of postoperative glaucoma between the two conditions was not significantly different (P = 0.072).

### Role of primary IOL in bilateral congenital cataract

**Fig 3:**
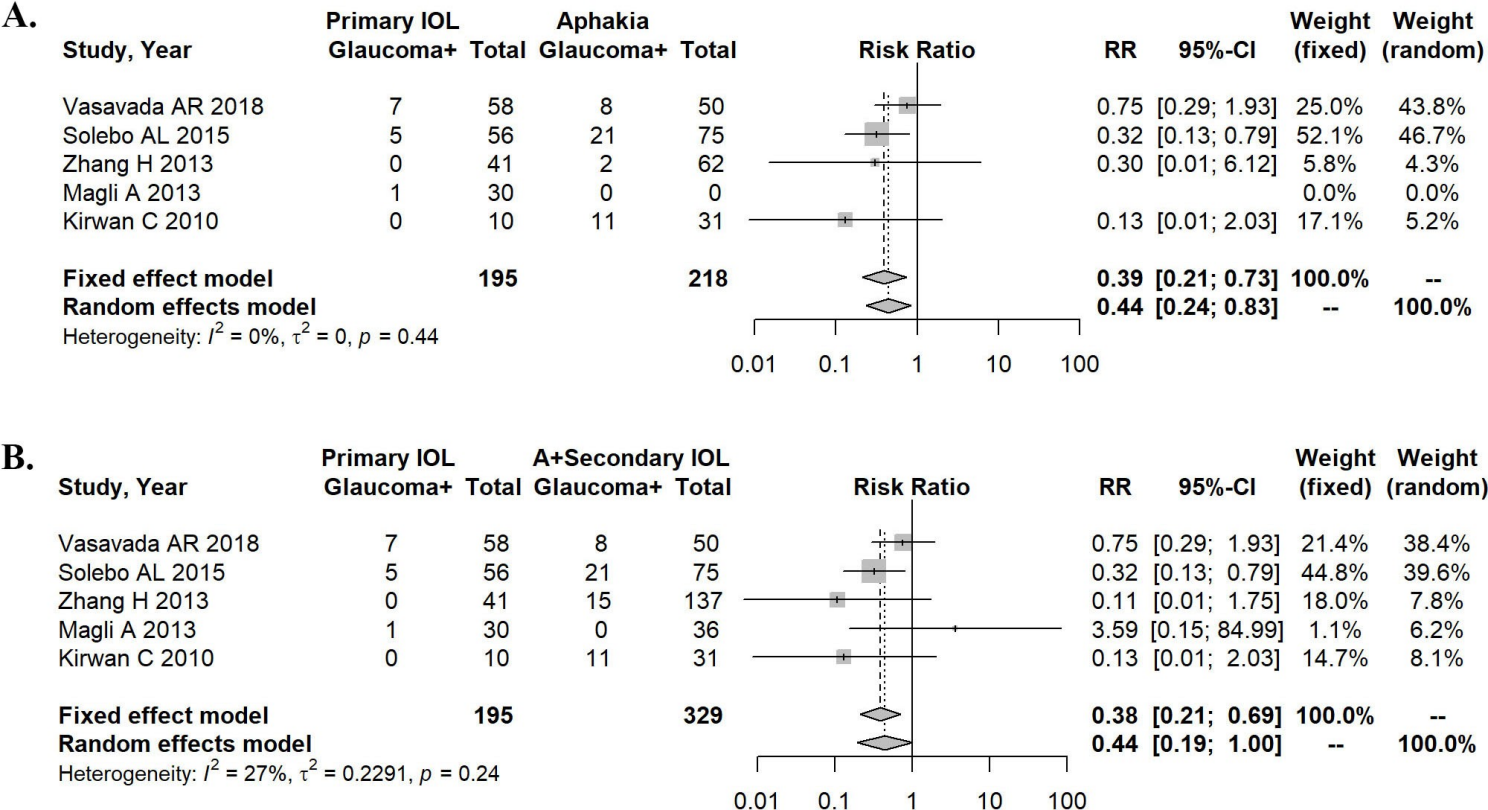
Forest plots describing the effects of treatments in patients with bilateral congenital cataract. A. Primary IOL implantation versus aphakia alone regarding the development of secondary glaucoma. B. Development of secondary glaucoma in primary IOL implantation versus aphakia and secondary IOL implantation.

We analyzed the data also for the subpopulation with bilateral congenital cataract. The heterogeneity of effect size was 0% for primary IOL in comparison with aphakia alone (P = 0.44). A random effects model was employed for the meta-analysis (Fig 3A), which favored primary IOL (RR = 0.44, 95% CI: 0.24–0.83). Moreover, a significantly lower incidence of postoperative glaucoma was determined for patients with primary IOL (P = 0.016).

When comparing primary IOL with aphakia and secondary IOL in bilateral congenital cataract, the heterogeneity of effect size was 27% (P = 0.24). The results of a random effects model used for the meta-analysis (Fig 3B) favored again primary IOL (RR = 0.44, 95% CI: 0.19–1.00). Moreover, patients with primary IOL had a significantly lower incidence of postoperative glaucoma (P = 0.042).

### Role of primary IOL in unilateral congenital cataract

**Fig 4:**
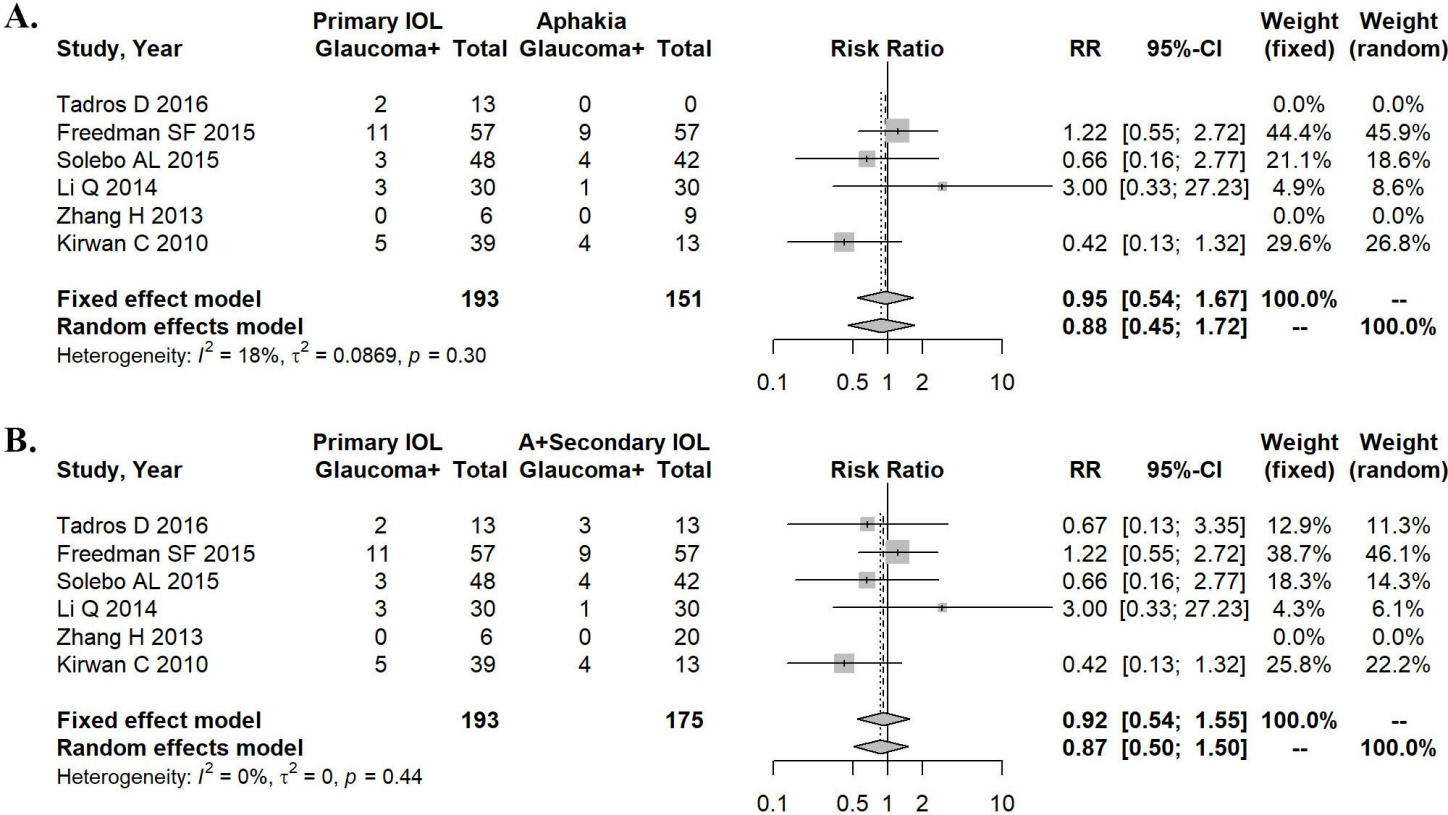
Forest plots describing the treatment effects in subpopulations with unilateral congenital cataract. A. Development of secondary glaucoma in primary IOL implantation versus aphakia alone. B. Primary IOL implantation versus aphakia and secondary IOL implantation regarding the development of secondary glaucoma.

In addition, we analyzed the data of patients with unilateral congenital cataract. In this population, the heterogeneity of effect size was 18% for primary IOL compared with aphakia alone (P = 0.30). A random effects model was used for the meta-analysis (Fig 4A), and no statistically significant difference was found in the incidence of postoperative glaucoma between these two conditions (RR = 0.88, 95% CI: 0.45–1.72, P = 0.710).

When comparing primary IOL with aphakia and secondary IOL in patients with unilateral congenital cataract, the heterogeneity of effect size was 0% (P = 0.44). The random effects model used for meta-analysis is shown in Fig 4B. The incidence of postoperative glaucoma was not statistically different between the two conditions (RR = 0.87, 95% CI: 0.50–1.50, P = 0.612).

### Publication bias

Funnel plots and Egger tests demonstrate the lack of publication bias for any of the analyses (S1–S3 Figs).

## Discussion

There are several therapeutic options for infant patients with congenital cataract after cataract extraction surgery, including primary IOL implantation and short-term aphakia followed by secondary IOL implantation. With the improved biocompatibility of IOLs and advances in surgical instruments, the most commonly recommended treatment for children over 2 years of age is a combined surgery, i.e., cataract extraction plus primary IOL implantation, but it remains a controversial procedure for IOL implantation in children below the age of 2 years [24]. Both methods are safe and effective to prevent the onset of deprivation amblyopia [25]. However, they also have their specific advantages and disadvantages. From this perspective, it is of great clinical significance to determine, whether primary IOL in congenital cataract patients under 2 years of age is the optimal approach to prevent or minimize the occurrence of secondary glaucoma.

Secondary glaucoma after congenital cataract surgery can be divided into early-stage glaucoma and late-stage glaucoma, based on the onset time. Most early-stage glaucoma present as angle-closure glaucoma resulting from vitreous pupillary block or inflammation of the peripheral anterior synechiae. With the development of advanced pediatric cataract surgery technologies and the appropriate postoperative usage of anti-inflammatory drugs, early-stage secondary glaucoma is rarely seen nowadays. Therefore, the studies included in this meta-analysis described mainly late-stage glaucoma, most of which were followed up for at least 5 years. Some researchers conclude that late-stage glaucoma frequently occurs as secondary open-angle glaucoma. The mechanism is unknown but may be related to trabeculitis [26] or toxicity of substances released from the vitreous [27].

To directly compare the incidence of secondary glaucoma in populations with and without primary IOL implantation, we conducted a meta-analysis using data of patients after primary IOL implantation and of patients with maintained aphakia or with secondary IOL implantation. Our aim in this study was to evaluate the risk of secondary glaucoma in patients who underwent different interventions; therefore, the incidence of secondary glaucoma was our exclusive outcome parameter. Although there are slight differences in the diagnostic criteria across the studies, they all comply with the consensus of Glaucoma diagnosis. The incidence of secondary glaucoma after congenital cataract surgery varies among reports and ranges from 6–24%, with follow-up periods of 45–66 months [28,29]. In accordance with previously reported data, the incidence of secondary glaucoma in primary IOL implantation is in our study 9.5% compared to 15.1% in aphakia and secondary IOL implantation. Our results demonstrate that the secondary glaucoma incidence after primary IOL implantation does not differ from that after aphakia and secondary IOL implantation in both the general cohort and the subpopulation with unilateral cataract. However, for bilateral cataract surgery, a significantly lower risk for postoperative glaucoma development was found in children with primary IOL implantation. Although the underlying mechanisms are unclear, the possible reasons are as follows. First, in patients with primary IOL implantation, the operation could prevent meshwork collapse and isolate the trabecular meshwork from the vitreous, since the vitreous will cause a toxic reaction when it contacts the meshwork [30]. Second, primary IOLs are commonly implanted into the capsular bag, whereas secondary IOLs are implanted in the ciliary sulcus. The latter procedure may lead to an enhanced inflammatory response in the anterior chamber angle [31]. Third, operations performed in several stages might cause increased inflammation [18]. Forth, early surgery in newborn infants has been reported to inhibit the eyeball growth [32] and interfere with the maturation of the trabecular meshwork [33], resulting in an increased risk of open-angle glaucoma. All these reasons increase the incidence of secondary glaucoma in secondary IOL implantation compared to that in primary IOL implantation. Additionally, spectacles and contact lenses used for aphakia have several drawbacks, including inconvenience, non-compliance, high cost, and increased risk of ocular infection and corneal injury [34].

The principle of treatment for congenital cataract should be to rehabilitate vision, to prevent or reduce amblyopia and blindness. It would be better to perform cataract surgery as early as possible in children with congenital cataract because 2 to 4 months after birth is in infants the critical period for the development of the fixation reflex [25]. Although studies have shown that younger patients usually have a higher incidence of secondary glaucoma, in addition to the fact that eyeballs of children are fragile and more susceptible to inflammation and intraocular pressure (IOP) variations [35], it is unnecessary to delay surgery only to reduce the risk of secondary glaucoma. In case of an increased IOP, ophthalmologists should start the treatment with anti-hypertensive eye drops, followed by surgery if necessary. It should be taken into consideration that a delayed operation may result in irreversible vision loss such as amblyopia. Fortunately, widely used vision screeners allow doctors to detect risk factors for amblyopia and intervene earlier, thereby reducing the incidence of amblyopia [36,37]. Patients with other types of congenital cataracts, that do not impair the vision seriously, such as anterior, coronal, or punctate cataract, should only be followed-up closely instead of immediate surgery.

In summary, the incidence of secondary glaucoma is variable and depends on the age at the time of surgery, on the primary or secondary IOL implantation method, on the ocular anatomy, and on the follow-up duration. Therefore, we ophthalmologists need to define personalized treatment strategies for patients with congenital cataract. In addition, it is important in clinical practice to detect IOP periodically during a long-term follow-up, because early intervention is the most effective way to reduce the risk of secondary glaucoma. Meanwhile, further studies are needed to better define the timing and approaches of the surgical procedures that can reduce the incidence of secondary glaucoma.

## Limitations

The limitations of the present meta-analysis should also be mentioned. First, we excluded several published studies that focused only on one treatment type for congenital cataract. Even though the incidences of secondary glaucoma are not statistically different between the general cohort with cataract surgery and the cohort with unilateral surgery, lower incidences were observed in patients with bilateral surgery. These observed differences may be rooted in the small sample size. We may get more conclusive results if we could include more patients. Second, there is a great variability of the mean follow-up time across the included studies, which may affect the prevalence of secondary glaucoma from study to study. Third, the operation methods were only described by the studies as cataract extraction. However, the surgical procedures lack standardization in our publication. Further investigations are needed to identify this kind of information in detail. Forth, several publications were excluded because full-text versions were not available; these studies might have provided important additional information.

## Supporting information

S1 Table PRISMA checklist 2009. (PDF)

S1 Fig Funnel plots describing the treatment effects in all congenital cataract patients. A. Primary IOL implantation versus aphakia regarding the development of secondary glaucoma. No publication bias was found (Egger test, P = 0.713). B. Primary IOL implantation versus aphakia and secondary IOL implantation regarding the development of secondary glaucoma. No publication bias was found (Egger test, P = 0.611).
(JPG)

S2 Fig Funnel plots describing the effects of treatments in patients with bilateral congenital cataract. A. Primary IOL implantation versus aphakia regarding the development of secondary glaucoma. No publication bias was found (Egger test, P = 0.504). B. Development of secondary glaucoma in primary IOL implantation versus aphakia and secondary IOL implantation. No publication bias was found (Egger test, P = 0.829).
(JPG)

S3 Fig Funnel plots describing the effects of treatments in patients with unilateral congenital cataract. A. Development of secondary glaucoma in primary IOL implantation versus aphakia alone. No publication bias was found (Egger test, P = 0.710). B. Primary IOL implantation versus aphakia and secondary IOL implantation regarding the development of secondary glaucoma. No publication bias was found (Egger test, P = 0.989).
(JPG)
