# Supplementary figures and images for: The role of primary intraocular lens implantation in the risk of secondary glaucoma following congenital cataract surgery: A systematic review and meta-analysis
Source: PLoS One. 2019 Apr 1;14(4):e0214684. doi: 10.1371/journal.pone.0214684 (PMC6443152; doi:10.1371/journal.pone.0214684)

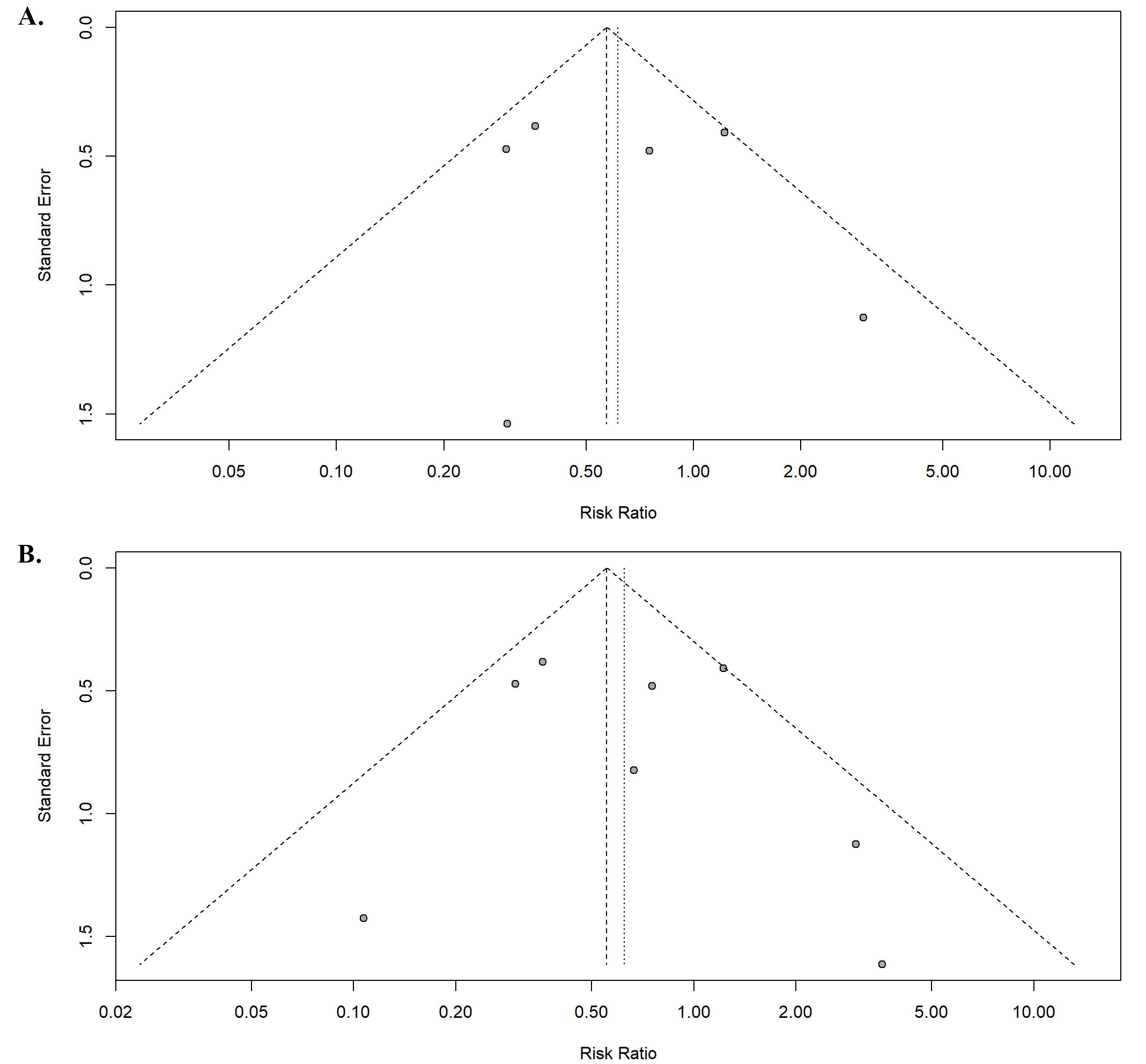

Supplement: S1 Fig — A. Primary IOL implantation versus aphakia regarding the development of secondary glaucoma. No publication bias was found (Egger test, P = 0.713). B. Primary IOL implantation versus aphakia and secondary IOL implantation regarding the development of secondary glaucoma. No publication bias was found (Egger test, P = 0.611). (JPG) [file pone.0214684.s002.jpg]

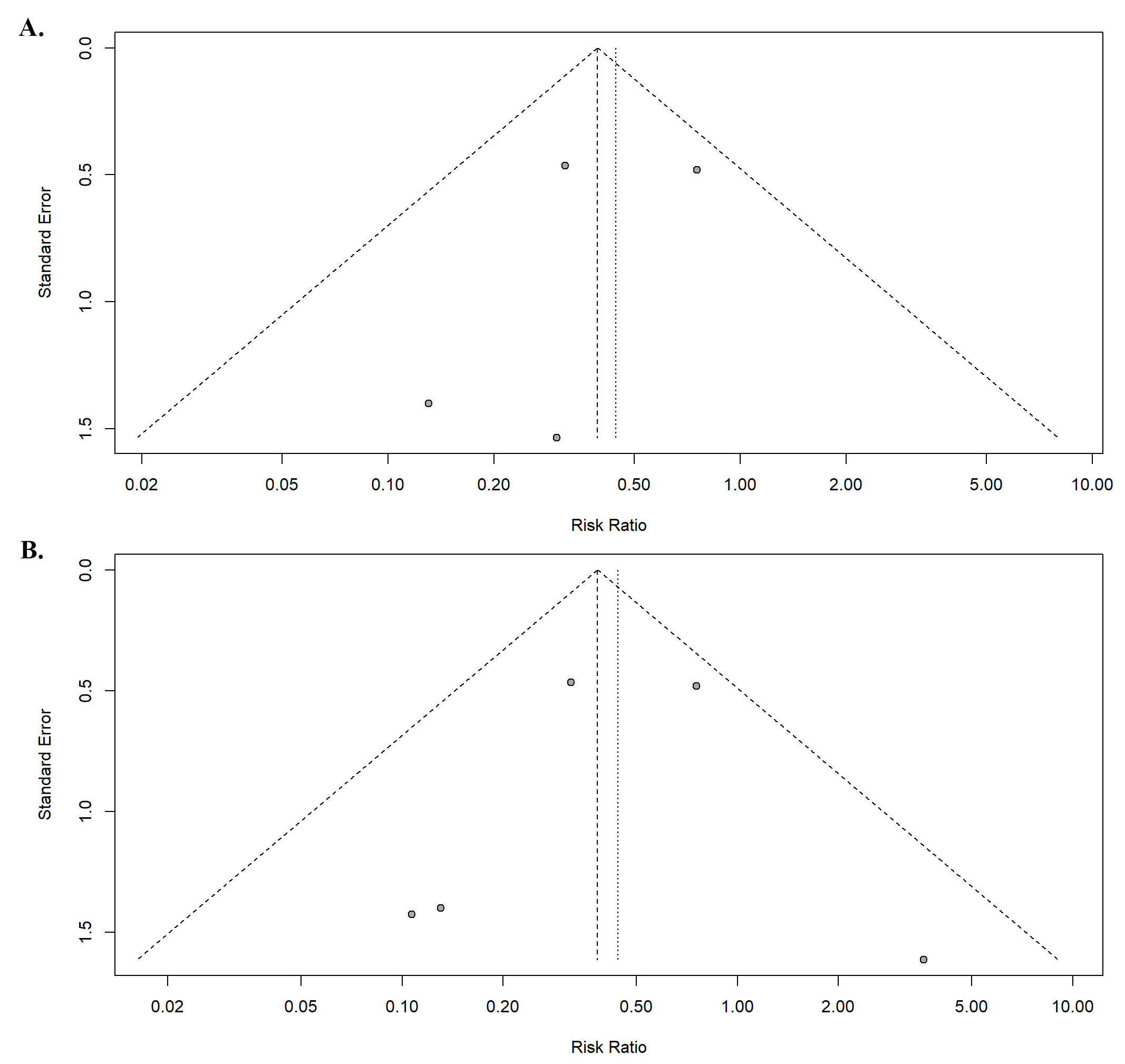

Supplement: S2 Fig — A. Primary IOL implantation versus aphakia regarding the development of secondary glaucoma. No publication bias was found (Egger test, P = 0.504). B. Development of secondary glaucoma in primary IOL implantation versus aphakia and secondary IOL implantation. No publication bias was found (Egger test, P = 0.829). (JPG) [file pone.0214684.s003.jpg]

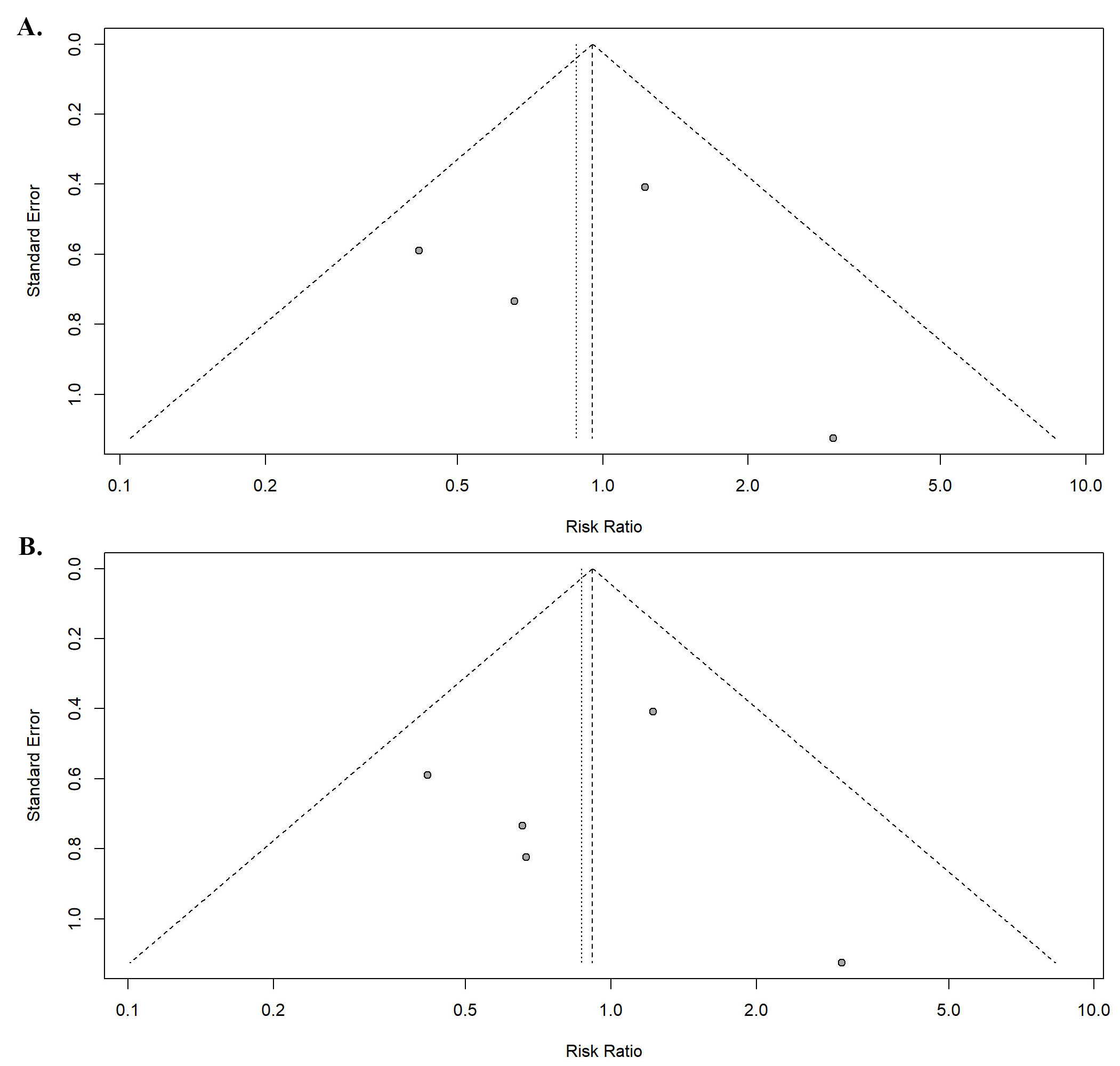

Supplement: S3 Fig — A. Development of secondary glaucoma in primary IOL implantation versus aphakia alone. No publication bias was found (Egger test, P = 0.710). B. Primary IOL implantation versus aphakia and secondary IOL implantation regarding the development of secondary glaucoma. No publication bias was found (Egger test, P = 0.989). (JPG) [file pone.0214684.s004.jpg]
